# Morphometric Characterization of Rat and Human Alveolar Macrophage Cell Models and their Response to Amiodarone using High Content Image Analysis

**DOI:** 10.1007/s11095-017-2176-5

**Published:** 2017-05-24

**Authors:** Ewelina Hoffman, Aateka Patel, Doug Ball, Jan Klapwijk, Val Millar, Abhinav Kumar, Abigail Martin, Rhamiya Mahendran, Lea Ann Dailey, Ben Forbes, Victoria Hutter

**Affiliations:** 1 Centre for Topical Drug Delivery and Toxicology School of Life and Medical Sciences, University of Hertfordshire, Hatfield, Hertfordshire, AL10 9AB UK; 2 Department of Pharmaceutical Biochemistry and Molecular Diagnostics Pharmacy Faculty, Medical University of Lodz, 1 Muszynskiego Street, 90-151 Lodz, Poland; 3 Institute of Pharmaceutical Science, King’s College London, Franklin-Wilkins Building, 150 Stamford Street, London, SE1 9NH UK; 4 Sackler Institute of Pulmonary Pharmacology, Institute of Pharmaceutical Science, Faculty of Life Sciences and Medicine, King’s College London, 150 Stamford Street, Waterloo Campus, London, SE1 9NH UK; 5 Medicines Research Centre, Glaxo-Smith-Kline, Gunnels Wood Road Stevenage, Hertfordshire, SG1 2NY UK; 6 GE Healthcare Life Sciences, Maynard Centre, Forest Farm, Whitchurch, Cardiff, CF14 7YT UK; 7 Institute of Pharmaceutical Technology and Biopharmacy, Martin Luther University Halle-Wittenberg, Wolfgang-Langenbeck-Str. 4, 06108 Halle (Saale), Germany

**Keywords:** foamy macrophage, NR8383, toxicology, U937, vacuolation

## Abstract

**Purpose:**

Progress to the clinic may be delayed or prevented when vacuolated or “foamy” alveolar macrophages are observed during non-clinical inhalation toxicology assessment. The first step in developing methods to study this response *in vitro* is to characterize macrophage cell lines and their response to drug exposures.

**Methods:**

Human (U937) and rat (NR8383) cell lines and primary rat alveolar macrophages obtained by bronchoalveolar lavage were characterized using high content fluorescence imaging analysis quantification of cell viability, morphometry, and phospholipid and neutral lipid accumulation.

**Results:**

Cell health, morphology and lipid content were comparable (*p* < 0.05) for both cell lines and the primary macrophages in terms of vacuole number, size and lipid content. Responses to amiodarone, a known inducer of phospholipidosis, required analysis of shifts in cell population profiles (the proportion of cells with elevated vacuolation or lipid content) rather than average population data which was insensitive to the changes observed.

**Conclusions:**

A high content image analysis assay was developed and used to provide detailed morphological characterization of rat and human alveolar-like macrophages and their response to a phospholipidosis-inducing agent. This provides a basis for development of assays to predict or understand macrophage vacuolation following inhaled drug exposure.

## Introduction

Airway diseases such as asthma and chronic obstructive pulmonary disease (COPD) are an area of unmet clinical need despite considerable investment in developing new therapeutics in this area [1, 2]. New inhaled medicines require extensive non-clinical safety assessment before progressing to the clinic for evaluation of safety and efficacy in humans [1–6]. It is not uncommon for alveolar macrophage responses to be observed in histological lung slices of animals during non-clinical inhalation toxicology studies: these are typically characterised by a highly vacuolated appearance and larger cell size [2, 3]. These cell responses may be unaccompanied by other changes or may be associated with other immune cell infiltration or remodelling of lung tissue [2, 3]. As the mechanism for induction of this alveolar macrophage phenotype and its relation to lung pathophysiology are not well understood, safe exposure levels are set without knowing whether these observations are truly an adverse response or an adaptation to high doses [2–4]. Due to these concerns over safety, inhaled compounds may fail in human studies due to lack of efficacy at the doses permitted [3–5].

“Foamy macrophage” is a term used by pathologists to describe an alveolar macrophage with a vacuolated cytoplasm when viewed by light microscopy [1, 2]. Currently, there are no universally accepted criteria to differentiate whether this vacuolated appearance constitutes an adaptive (non-adverse) or an adverse response. It is hypothesized that inhaled drugs may induce the foamy appearance in alveolar macrophages via different pharmacological and non-pharmacological mechanisms [7–10]. For example, a vacuolated appearance may be an appearance common to a reversible response due to the uptake of poorly soluble inhaled drug particulates or indicative of an inimical disruption of metabolic activity [1–4].

The need to understand macrophage responses mechanistically and develop techniques to identify ‘problem’ molecules early during development and discriminate these from agents that induce non-adverse macrophage responses is well recognized [3]. Validated tools with this capability would improve the efficiency of drug development process by identifying poor drug candidates early and removing barriers to the clinical translation of good drug candidates. Such a scientific, evidence-based approach would also align with 3R principles and reduce the burden of animal testing by reducing the need to perform or repeat *in vivo* toxicology studies, especially if *in vitro* methodologies can be developed. *In vitro* strategies for screening new drug compounds for their propensity to induce a macrophage vacuolation and study the mechanistic basis of the vacuolated state have been recently reported [7]. Discerning the different mechanisms by which the vacuolated phenotypes develop and resolve is key to understanding the safety implications of this phenomenon.

The high content analysis screen proposed recently by Hoffman *et al*. utilized fluorescence imaging of untreated and drug-treated murine macrophages to generate morphometric data (cell and nuclear area, vacuole number, vacuole size) alongside cell viability and functionality data (mitochondrial activity, membrane permeability, phagocytosis, activation state) [7]. This preliminary study was designed to compare the morphometric and functional properties of *in vitro* murine macrophages, J774A.1 cells in response to selected drug challenges [7].

The current study describes development of the fluorescence imaging methodology to obtain quantitative morphometric data across three cell types (rat and human cell lines *versus* primary rat alveolar macrophages). Early *in vivo* pre-clinical safety and efficacy studies for new inhaled drug candidates in the pharmaceutical industry are predominantly conducted in the rat [1–3]. The NR8383 rat macrophage cell line is well established and is widely used for *in vitro* inhalation toxicity prediction studies [8–10]. In the absence of a specific human alveolar macrophage cell line being commercially available, U937 cells (human monocytes derived from a pleural effusion) were employed being the only human monocyte cell line to originate from the lung and were differentiated to macrophages using established protocols [11–16]. These cell sources were selected to represent rat and human species and make comparisons between the rat cell line and primary rat alveolar macrophages. The aim of the study was to develop the high content methodology reported previously [7], to include cell health, morphometric data and lipid profiling within the same 96-well format. The assay was used to characterize the two cell lines cultured using standard conditions reported in the literature and compare vacuolation profiles of the cells in the unperturbed state and following exposure to a well-known foamy macrophage inducer compound, amiodarone [17–29], for 24 and 48 h. Algorithms for converting images into morphometric data were developed and the most sensitive statistical parameters to reflect cell population responses were established. The results reported here document important information on the baseline condition of the cell lines, comparison with rat primary alveolar macrophages under the same assay conditions, and provide the basis from which to develop an *in vitro* predictive assay for vacuolated macrophage induction.

## Materials and Methods

### Cell Culture

Rat macrophage (NR8383) and human monocyte (U937) cell lines were purchased from LCG Standards (Teddington, Middlesex, UK) and used between passage 2 and 20 from purchase. NR8383 cells were cultured as described previously by others [8–10]. In brief, cells were cultured in Kaighn’s modified Ham’s F12 (K-F12) medium with 15% *v*/v heat-inactivated fetal bovine serum (FBS) and supplemented with 100 IU/ml penicillin-100 μg/ml streptomycin solution and 2 mM L-glutamine (Sigma Aldrich, Dorset, UK). U937 cells were cultured as previously described in literature [11–16]. In summary, cells were maintained in RPMI with 10% *v*/v FBS and supplemented with 100 IU/ml penicillin-100 μg/ml streptomycin solution and 2 mM L-glutamine (Sigma Aldrich, Dorset, UK). U937 cells were cultured in a humidified atmosphere at 37°C with 5% *v*/v CO_2_ and cell number was maintained between 1 x 10^5^ to 2 x 10^6^ cells/ml. For experiments, cells were seeded onto bottom μclear black 96-well plates (Greiner Bio-One, Gloucester, UK) at an optimal density of 3 x 10^4^ cells/well in 100 μl of complete cell culture medium. The cell density was determined experimentally to ensure equal cell distribution in the well for optimal image capture. U937 cells were differentiated to a macrophage phenotype using 4 nM phorbol myristate acetate (PMA) (Sigma Aldrich, Dorset, UK) in complete cell culture media for 96 h followed by a 24 h rest period in complete cell culture medium as previously described [16].

### Rat Alveolar Macrophages Obtained from Lavage

Male Wistar Han rats were supplied by Charles River between 7–9 weeks of age (approximately 250–300 g). Alveolar macrophages were obtained according to established methodology [28]. In brief, the trachea of naïve rats was isolated by a midline incision, cannulated and the lungs washed with 3 x 5 mL bronchoalveolar lavage (BAL) fluid (3.72 g ethylenediaminetetraacetic acid /1 g bovine serum albumin/L in phosphate buffered saline (PBS)). The individual aliquots from each animal were combined to form one BAL sample per animal and stored on wet ice. The samples were centrifuged at 250 *g* for 5 min at 4°C. Cells pellets were re-suspended in 1 mL of complete culture medium as outlined for U937 cells above. For experiments, primary alveolar macrophages cells were obtained from naïve rats after two timepoints (day 1 and day 7) of an *in vivo* study due to the limited quantity of alveolar macrophages available from BAL. No differences were anticipated between cells isolated on different days of the *in vivo* study. These cells were seeded onto bottom μclear black 96-well plates (Greiner Bio-One, Gloucester, UK) at a density of 1.5 x 10^4^ cells/well in 100 μL of complete cell culture medium. The plate was centrifuged at 380 *g* for 5 min at 20°C and the cells incubated for 2 h in a humidified atmosphere at 37°C with 5% *v*/v CO_2_ to allow macrophages to attach to the plates before undergoing the fluorescence staining procedure. To understand the impact of prolonged *in vitro* culture on primary rat alveolar macrophages, cells from naïve rats on day 28 of an *in vivo* study were harvested and cultured *in vitro* on 96-well plates for 24 and 48 h prior to analysis.

### Fluorescence Staining and Imaging

Cells were either incubated with amiodarone (0.03–100 μM) in complete cell culture medium with 1% *v*/v DMSO for 24 and 48 h alongside internal controls of either untreated cells, 1% *v*/v DMSO vehicle control, 200 μM carbonyl cyanide-4-(trifluoromethoxy)phenylhydrazone (FCCP) mitochondrial activity control or 0.5% *v*/v Triton X-100 (TX-100) cell death control. Compound exposure studies were conducted 24 h after seeding (NR8383) or after differentiation (U937) for cell lines. For primary macrophages, cells were exposed to compounds 2 h after isolation once the cells had attached to the 96-well plate. For cell health and morphology assessment, cells were stained with a dye cocktail containing Hoechst 33342 10 μg/mL, MitoTracker Red 300 nM and Image-It Dead Green 25 nM (Invitrogen, Renfrewshire, UK) for 30 min. Cells were washed once with 100 μL PBS and centrifuged at 380 g before fixation with 3.7% *w*/*v* paraformaldehyde for 15 min. Fixed cells were stained overnight with Cell Mask Deep Red (Invitrogen, Renfrewshire, UK) diluted 1:1000 (according to the manufacturer’s protocol). Cells were washed once with PBS as described above before imaging. For the determination of lipid content, cells were incubated with HCS LipidTox Phospholipid Red (Invitrogen, Renfrewshire, UK) diluted 1:1000 (according to the manufacturer’s protocol) for 24 h or 48 h. Cells were fixed with 3.7% *w*/*v* paraformaldehyde containing Hoechst 33342 (10 μg/mL) for 20 min, followed by one wash with PBS. Cells were then incubated with HCS LipidTox Green (Invitrogen, Renfrewshire, UK) diluted 1:1000 (according to the manufacturer’s protocol) for 30 min at room temperature for detection of neutral lipids. Cells from both assays were stored at 4°C before sample acquisition. Images were captured using the In Cell Analyser 6000 (GE Healthcare, Little Chalfont, Bucks, UK) with a 40x objective in standard 2D imaging mode with an exposure time of 0.1 s.

### Quantitative High Content Analysis

Image analysis was performed using In Cell Developer Toolbox v 1.9.2, Level 3 analysis (GE Healthcare, Little Chalfont, Bucks, UK). For cell health and morphology analysis, Hoechst 33,342 cell nuclear staining was used to identify nucleated cells. Cell Mask Deep Red dye is an established cell delineation tool for cellular imaging and was used to highlight the cytoplasmic regions within the cells identified. Conversely, vacuoles within cells were identified based on negative staining with Cell Mask Deep Red. MitoTracker Red detects the changes in the mitochondrial membrane potential and accumulates in active mitochondria. Image-It Green Dead is an impermeant dye to healthy cells that becomes permeant when the plasma membrane of cells is compromised. These two cell health stains were reported as fluorescence intensity values.

For lipid content analysis, Hoechst 33342 was used to segment the cell nuclei as described above and HCS LipidTox Green was used to identify the cell area. The intercellular accumulation of phospholipids (phospholipidosis) was detected and quantified by cells incubation with phospholipids conjugated to fluorescent dye – LipidTox Phospholipidosis Red. The intercellular accumulation of neutral lipids (steatosis) was detected by LipidTox Green Neutral Lipids Dye, which has high affinity for neutral lipids. Both stains were reported as fluorescence intensity values.

Eight quantitative measurements were generated from the analysis, namely cell area, nuclear area, mitochondrial activity, cell permeability, vacuole number per cell, vacuole area per cell, phospholipid content per cell and neutral lipid content per cell which characterized cell health, morphology and lipid content. Data represented 9–12 different experiments, *n* = 6 wells per experiment, for untreated rat and human cell lines and 3 different experiments, *n* = 1 well per experiment, for amiodarone-exposed cells in 3 different experiments. Equivalent experiments were performed for primary rat alveolar macrophages cultured *in vitro* for 24 and 48 h from the BAL of 3 rats, with *n* = 6 wells per animal. Rat alveolar macrophages obtained from BAL were also assessed after 2 h of attachment in a 96-well plate without further *in vitro* culture from 3 different rats with *n* = 3 wells per animal.

### Secondary Analysis Criteria: Exclusion of Cells with Reduced Mitochondrial Activity

**Fig. 1:**
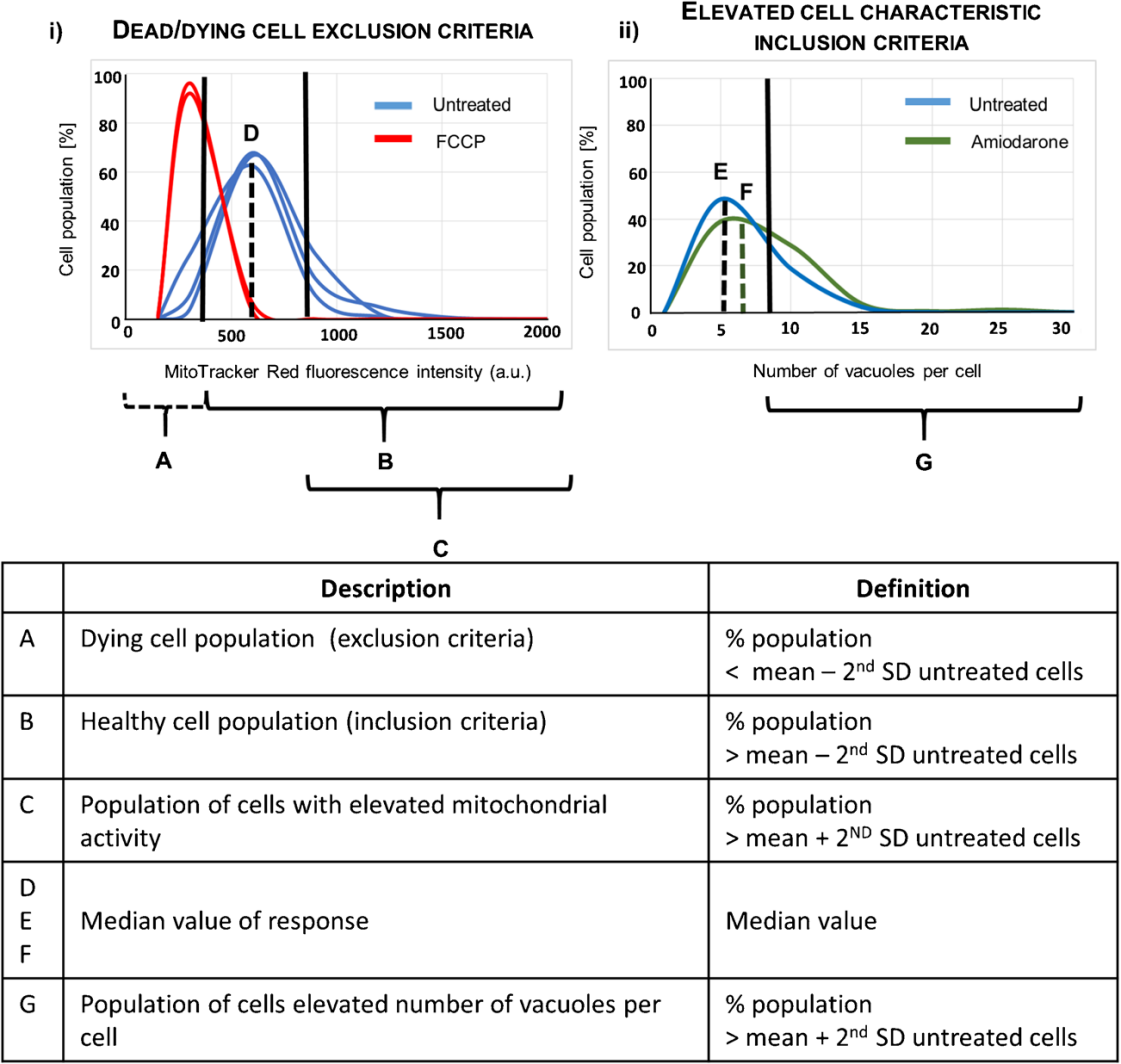
Representation of data analysis approach. Single cell data was summarised as a frequency histogram to assess the reproducibility of untreated cells, control treatments and sample treatments (i) Dying cells (defined as the population with a mitochondrial activity < mean value minus 2nd SD) were excluded from the analysis (**a**), and the remainder of the cell population were included (**b**). Initial data analysis compared the median response values (**d**, **e**, **f**). Elevated cell responses (**c** and **g**) defined as the population of cells with cell characteristic > mean plus the 2nd SD from the untreated cell population were also calculated and data summarised as elevated response profiles (ii).

Criteria to identify dead and/or dying cells were defined so that these cells could be excluded from the analysis to prevent morphology and lipid characterization parameters being impacted by changes induced from cell death. Exposure conditions that reduced the total number of cells imaged to less than 50% of the number of cells image in control (untreated) cells were excluded. In the remaining wells, cells with reduced mitochondrial activity were classified as those possessing less than two standard deviations below the mean fluorescence intensity of MitoTracker Red dye in the untreated cell population and were excluded from the analysis (Fig. 1). Mitochondrial activity was selected as a more sensitive marker over cell permeability as the washing steps within the assay methodology removed the majority of cells with compromised cell membrane integrity. Cells exposed to 200 μM FCCP (potent mitochondrial oxidative phosphorylation uncoupling agent) were used as an internal assay control to confirm that the mitochondrial activity exclusion boundary set was appropriate. Cell viability has been measured as percentage of cells with mitochondrial activity above the mean fluorescent value minus the second standard deviation, out of total number of cells per well.

### Statistical Analysis

Morphology and lipid content median data from repeated experiments were assessed using the Shapiro-Wilk test for normality and found to fit a normal (Gaussian) distribution. One-way ANOVA analysis with Tukey-Kramer multiple comparison post-hoc tests were used to assess the statistical significance between the different cell models. Statistical significance was evaluated at a 95% confidence level (*p* < 0.05). All statistical tests were performed using Graphpad Instat® version 3.06.

## Results

### Cell Morphology and Lipid Content: Average Population Data

**Fig. 2:**
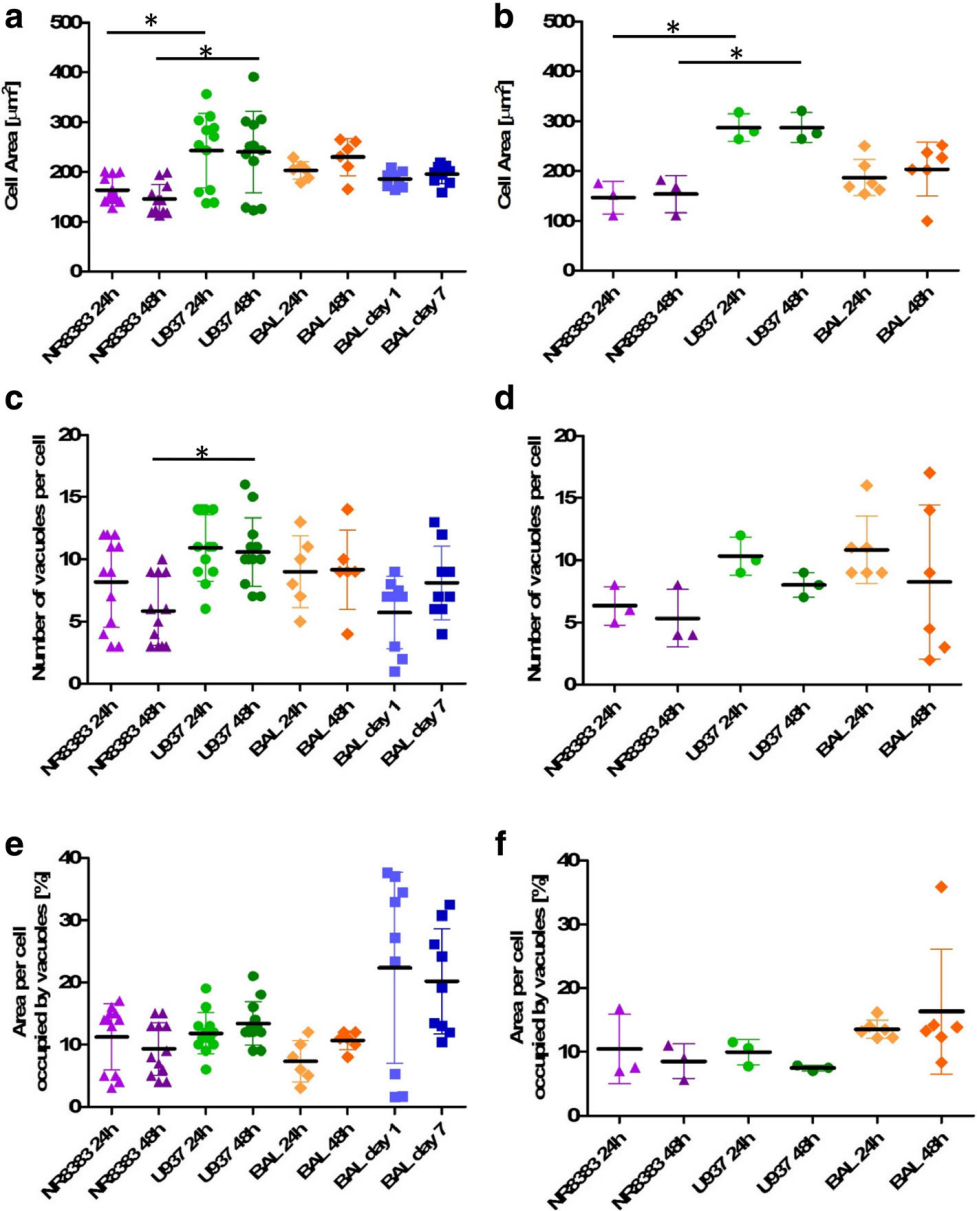
Median morphology parameters of untreated and amiodarone-treated rat and human macrophage cell models. Median cell area, vacuole number per cell and percentage of cell area occupied by vacuoles for untreated cells (**a**, **c**, **e**) and cells exposed to 10 μM amiodarone (**b**, **d**, **f**). NR8383 rat macrophage cells (purple triangle), U937 human monocyte-derived macrophage cells (green circles), primary rat macrophages derived from BAL in in vitro culture for 24 h or 48 h (orange diamonds) and primary rat macrophages derived from BAL tested immediately after harvesting from naïve rats after 1 and 7 day timepoints (blue squares). Each data point represents the median value of *n* = 6 wells per plate. * indicates *p* < 0.05.

**Fig. 3:**
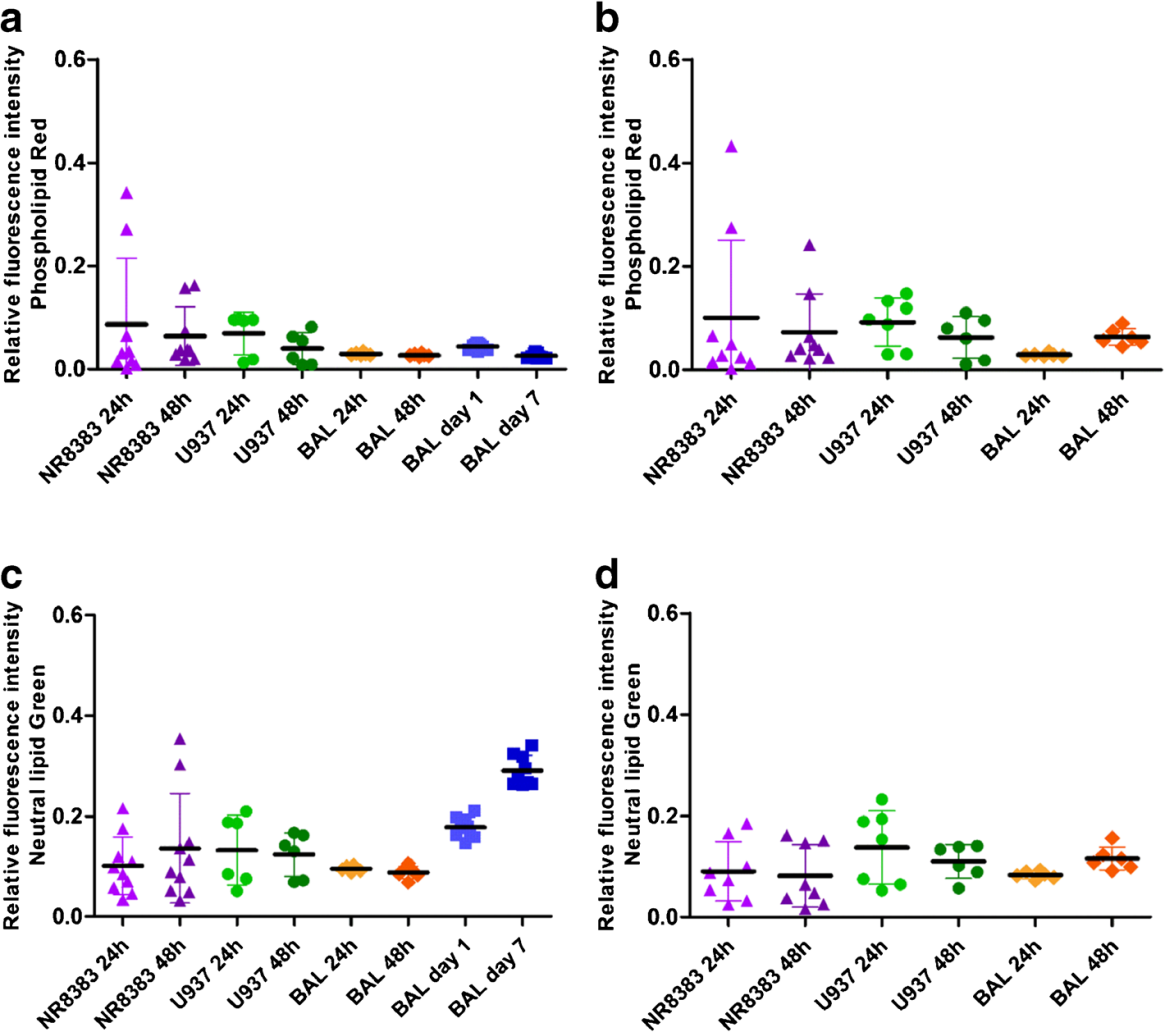
Median lipid parameters of untreated vs amiodarone-treated rat and human macrophage cell models. Median phospholipid content and neutral lipid content for untreated cells (**a, c**) and cells exposed to 10 μM amiodarone (**b, d**). NR8383 rat macrophage cells (purple triangle), U937 human monocyte-derived macrophage cells (green circles), primary rat macrophages derived from BAL in in vitro culture for 24 h or 48 h (orange diamonds) and primary rat macrophages derived from BAL tested immediately after harvesting from naïve rats after 1 and 7 day timepoints (blue squares). Each data point represents the median value of *n* = 6 wells per plate. * indicates *p* < 0.05.

Individual cell data for each parameter derived from the images were plotted and morphology and lipid content histograms assessed for normality (Fig. 1). As not all data were found to fit a normal Gaussian distribution, median values were obtained for all parameters and used to compare the different macrophage cell types and the effect of exposure to amiodarone (Figs. 2 and 3).

Cell morphology was reproducible in each of the models under investigation. The data ranged from 143–262 µm^2^ median cell area, 6–11 vacuoles per cell and 7–27% area occupied by vacuoles in all cell models investigated (Fig. 2). No significant differences (*p* > 0.05) were observed for morphology after 24 or 48 h in culture. Whilst a significant difference (*p* < 0.05) was observed for cell area between NR8383 and U937 cells for both time points tested, U937 cell area was not significantly different (*p* > 0.05) from rodent alveolar macrophages obtained from BAL. Median cell area was more variable for U937 cells (123–391 µm^2^) in contrast with the NR8383 cell line (113–201 µm^2^) or primary *in vivo* alveolar macrophages from BAL (165–265 µm^2^). This variability may be due to the PMA-mediated differentiation process undergone by the monocytic U937 cells to generate an alveolar macrophage phenotype. Vacuole number was also found to be significantly different (*p* < 0.05) between NR8383 and U937 cells (6 *versus* 10) cultured for 48 h but not between NR8383 and rat primary alveolar macrophages. Rat alveolar macrophages obtained from BAL from naïve rats after 1 and 7 days of an *in vivo* study and characterized immediately after a 2 h attachment period produced the most variable vacuolation. In contrast, BAL cells cultured for 24 and 48 h *in vitro* displayed more reproducible vacuole characteristics. Whilst intra subject variation for vacuole descriptors was low for freshly isolated primary rat alveolar macrophages, variability was greater between the 3 individual rodents assessed. Untreated cell median lipid content data was reproducible in all models tested (Fig. 3). No significant differences (*p* > 0.05) were observed for morphology descriptors between models with the same cell source after 24 or 48 h. Additionally, no significant differences (*p* > 0.05) were observed for either phospholipid or neutral lipid content between the different cell models investigated.

No significant difference (*p* > 0.05) was observed between untreated cells and those exposed to 10 μM amiodarone (known inducer of phospholipidosis) when comparing median values, despite observable differences in histogram profiles (Fig. 1). Average population statistical analysis was not a suitably sensitive descriptor to depict the morphology and lipid changes observed in cells exposed to amiodarone which impacted less than 20% of the total cell population. Therefore, an alternative analytical approach which included only the cell population with elevated characteristics in comparison with the untreated cells was considered.

### Macrophage Responses to Amiodarone: Elevated Vacuolization and Lipid Content

**Fig. 4:**
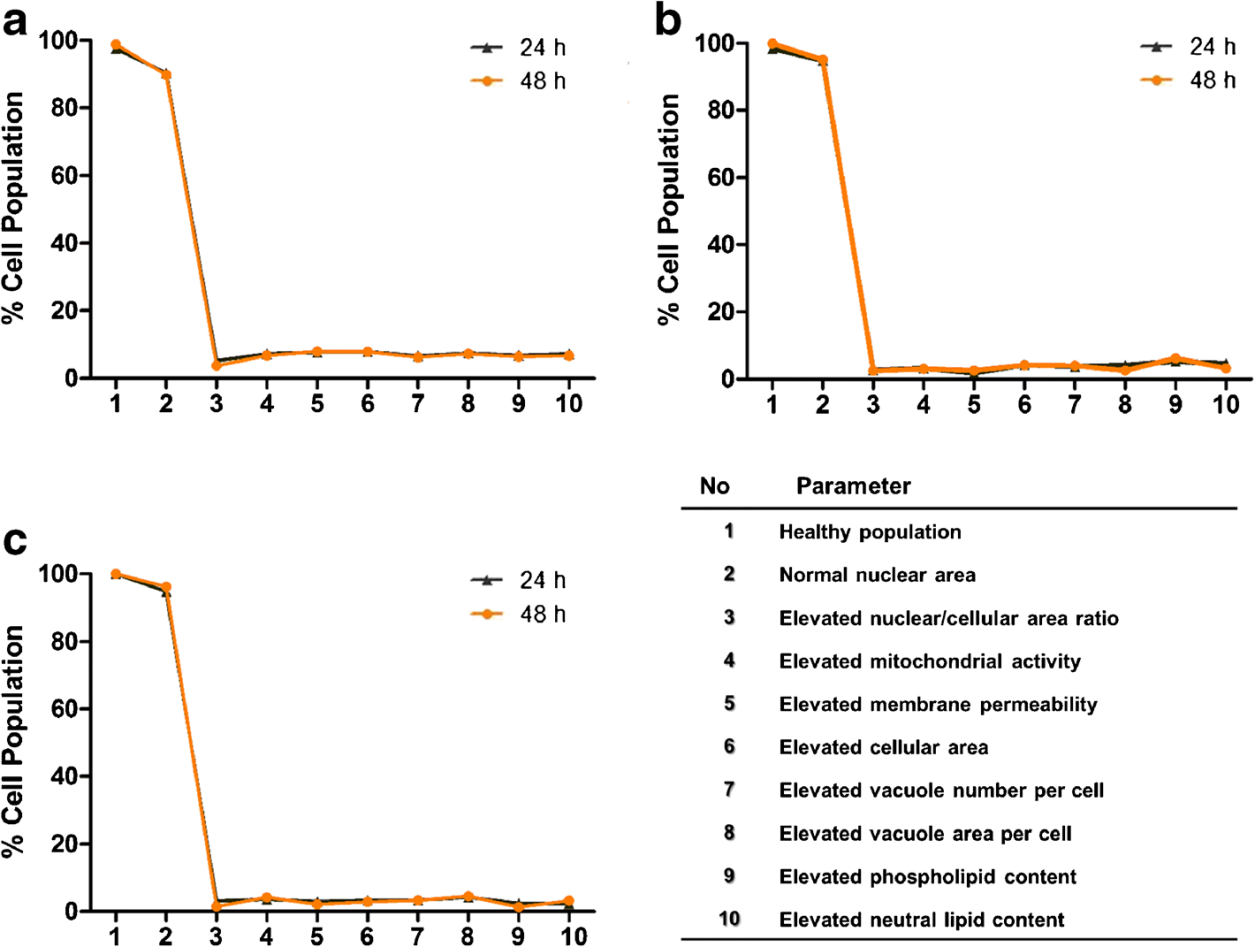
Cell health, morphology and lipid multi-parameter baseline profiles. Baseline multi-parameter charts profiling cell health, morphology and lipid changes to the NR8383 rat alveolar macrophage cell line (**a**), U937 human monocyte-derived macrophage cell line (**b**) and primary rat macrophages obtained from BAL (**c**) after 24 h (black line) and 48 h (orange line) timepoints. Data is represented as mean of 3 experiments where *n* = 6 for each investigation.

Profiles describing the cell population were generated to describe cell health, morphology and lipid content characteristics that were elevated from untreated cells. Dead/dying cells were eliminated as described above and mean nuclear area ± two standard deviations of the untreated cell population was used as an exclusion criteria to remove cells with partial or double nuclei (dividing cells). The remainder of cell health, morphology and lipid profile descriptors were classified as those that were elevated from untreated controls (Table I). ‘Elevated’ responses were defined as being greater than two standard deviations above the mean of the untreated control cell population (Fig. 1). The cell health inclusion criteria set resulted in >95% of all cells being included in the analysis and of these, >90% of cells had nuclear area within two standard deviations of the mean (Fig. 4). Analysis of untreated *in vitro* cultured U937, NR8383 and BAL-derived cells indicated that 5–8% of the population cell health, morphology and lipid content characteristics were considered ‘elevated’ at two standard deviations above the mean for both 24 and 48 h time points tested. Elevated cell responses were comparable for all cell types tested after 24 and 48 h (Fig. 4).

**Table I:** Summary of inclusion/exclusion criteria and elevated profile parameters

Parameter	Inclusion criteria
Healthy cell inclusion criteria	% cells > mean – 2nd SD mitochondrial activity
Normal nuclear area	% cells > mean nuclear area - 2nd SD and < mean + 2nd SD of the untreated cell population
Elevated mitochondrial activity	% cells > mean mitochondrial activity +2nd SD of the untreated cell population
Elevated membrane permeability	% cells > mean membrane permeability +2nd SD of the untreated cell population
Elevated cellular area	% cells > mean cell area + 2nd SD of the untreated cell population
Elevated nuclear/cellular area ratio	% cells > mean nuclear/cell area ratio + 2nd SD of the untreated cell population
Elevated vacuole number per cell	% cells > mean vacuole number per cell +2nd SD of the untreated cell population
Elevated vacuole area per cell	% cells > mean vacuole area per cell +2nd SD of the untreated cell population
Elevated phospholipid content	% cells > mean phospholipid content per cell +2nd SD of the untreated cell population
Elevated neutral lipid content	% cells > mean neutral lipid content per cell +2nd SD of the untreated cell population

**Fig. 5:**
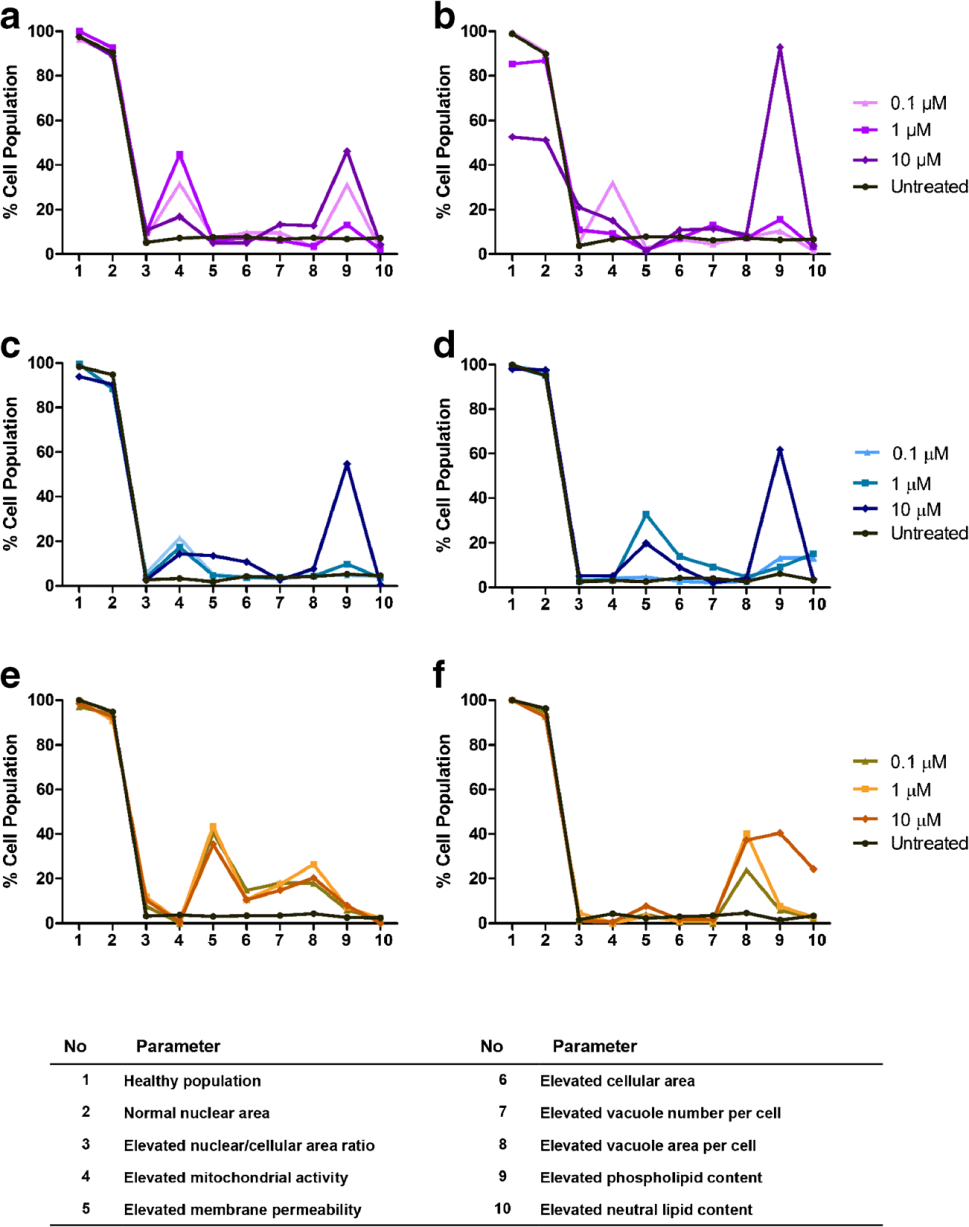
Cell health, morphology and lipid multi-parameter profiles after amiodarone exposure. Multi-parameter charts profiling cell health, morphology and lipid changes to the NR8383 rat alveolar macrophage cell line (**a**, **b**), U937 human monocyte-derived macrophage cell line (**c**, **d**) and primary rat macrophages obtained from BAL (**e**, **f**) after 24 h (**a**, **c**, **e**) and 48 h (**b**, **d**, **f**) exposure to 0–10 μM amiodarone. Data is represented as mean of 3 experiments where *n* = 6 for each investigation.

In contrast to median value analysis, multi-parameter profile analysis of the elevated population characteristics revealed an impact on elevated phospholipid content and cell health markers as expected for the phospholipidosis inducer, amiodarone (Fig. 5). A concentration dependent increase in the number of cells with elevated mitochondrial activity was observed for NR8383 and U937 cells after 24 h amiodarone exposure and NR8383 cells after 48 h exposure. Whilst elevated mitochondrial activity was not observed for U937 cells after 48 h exposure and BAL cells, enhanced membrane permeability was increased as a marker of impaired health for these cells. Enhanced phospholipid content was observed for all cells and time points investigated, and was more pronounced after 48 h exposure in all cell models. Perturbations in cell morphology, specifically elevated cell area, nuclear/cell area ratio, vacuole number and vacuole area per cell were observed for all three cell types tested. In general, these morphological changes were more pronounced after 24 h amiodarone exposure.

**Fig. 6:**
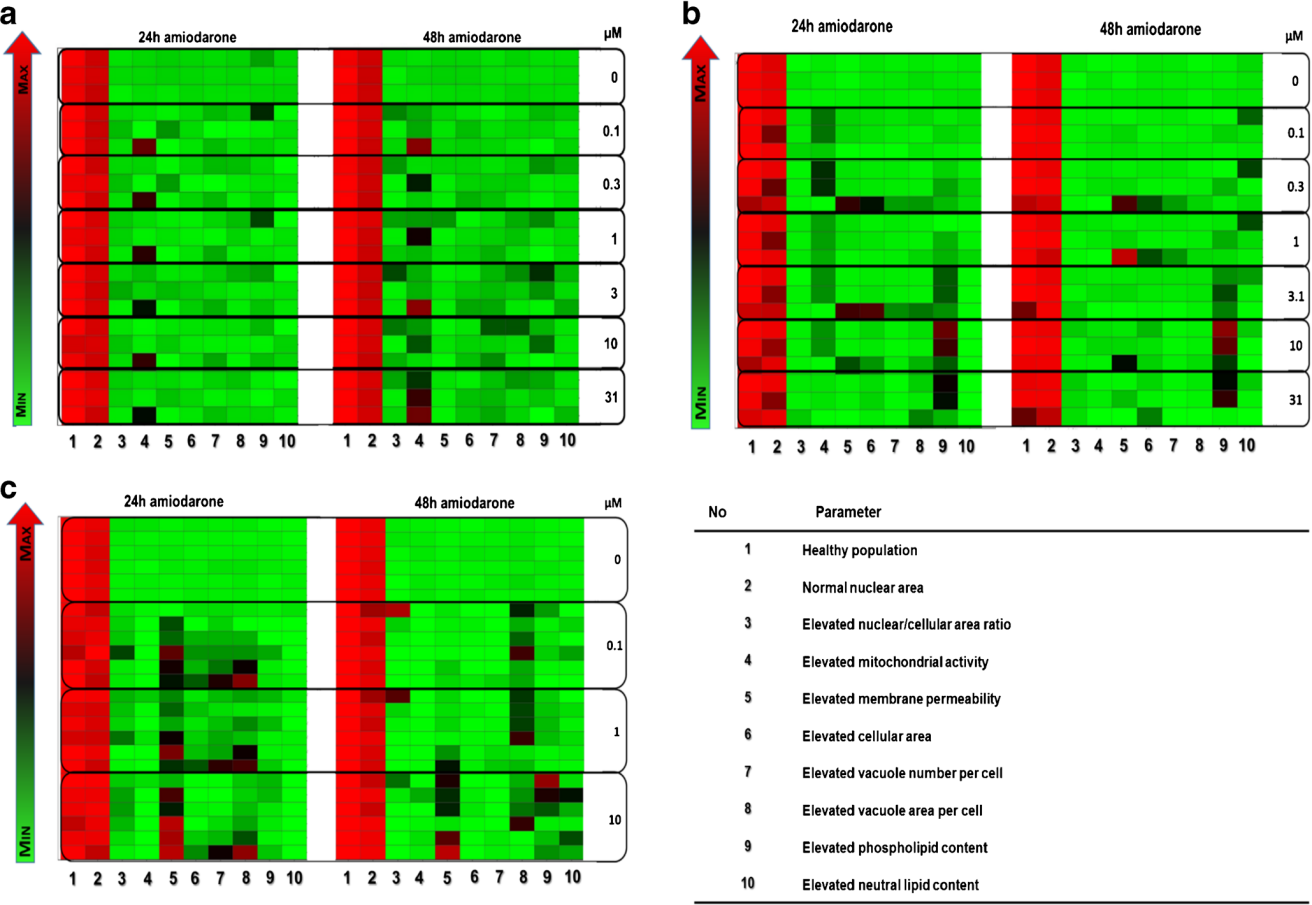
Heat map indicating experimental variability. Variability of profile parameters for cells exposed to 0–10 *μ*M amiodarone for 24 and 48 h for the NR8383 rat alveolar macrophage cell line (**a**), U937 human monocyte-derived macrophage cell line (**b**) and primary rat macrophages obtained from BAL (**c**). Each square represents one experiment. The colour gradient sets the lowest value for each given parameter in the heat map (bright green), highest value (bright red) and mid-range values (black) with a corresponding gradient between these extremes.

Heat maps generated to examine the reproducibility in terms of elevated parameters between cell passages and experiments indicated a high level of reproducibility in the untreated cell profile data for all three cell sources investigated (Fig. 6). Baseline profiles remained consistently reproducible for all macrophage cell sources tested and were not affected by passage number, time in culture or experimental variability.

## Discussion

The failure rate of inhaled compounds during drug development is costly to the pharmaceutical industry [29]. It is not known whether the foamy macrophage response (highly vacuolated appearance, increased cell size) observed in histological lung slices of rats from non-clinical *in vivo* studies are relevant to humans and safe exposure levels are set without knowing if these observations are truly adverse [2–4]. The foamy macrophage designation is applied as an umbrella term and is likely to cover a broad number of cell responses which manifest in a vacuolated appearance, but reflect different cell phenotypes with different impacts on airway pathophysiology [3].

Whilst *in vitro* approaches for predicting drug safety are used non-clinically in the pharmaceutical industry, multi-parameter screens combining viability, activation, functionality and morphology endpoints are not used routinely. Furthermore, morphological assessment by low resolution and confocal microscopy provides a crude description of the phenotype with little detail regarding the cellular changes [3]. Multi-parameter *in vitro* assessment of phenotypic alveolar macrophage responses may allow a more detailed understanding of pathological lung responses which can be correlated to morphological changes observed in the rat lung to help inform more detailed safety assessment.

This paper describes methodology for assessing changes in cell health, morphology and lipid content of *in vitro* and *ex vivo* alveolar macrophage cell models using a high content image analysis technique. Alveolar macrophage responses to drug challenge *in vivo* may impact only a small percentage of the total cell population [1, 2] and this was also observed in the *in vitro* studies. As a result, cellular responses *in vitro* may be overlooked when considering single, average data descriptors. The high content image analysis methodology described in this paper captures multiple parameters for individual cells, enabling analysis of a subset of the population of cells that respond to the drug stimulus. Amiodarone was used as a model drug to generate morphology and lipid profile responses consistent with the established phenotypic response of phospholipidosis it induces in alveolar macrophages [30–35].

Baseline profiles were established for the alveolar macrophage cell line NR8383, human PMA-differentiated monocyte cell line U937 and rat alveolar macrophages obtained from BAL immediately after isolation and after up to 48 h *in vitro* culture. With the exception of median cell area, untreated cells showed comparable cell morphology and lipid content parameters for both average and elevated cell response analysis methods between rat and human models tested. Cell characteristics investigated for all three *in vitro* models were highly reproducible and in accordance with observations from the macrophages obtained from the BAL of rats which had been exposed to air controls in inhalation toxicology study protocols, indicating their suitability for studying alveolar macrophage responses *in vitro*. As anticipated, morphometry was more variable between the different cell models after amiodarone treatment, however changes in cell health and phospholipid content were evident in all models as expected for cells exposed to a compound known to induce phospholipidosis. Further work is required to assess the variability in morphological response between different cell models and whether this reflects differences between species.

Interspecies differences in alveolar macrophage cell diameter between rats and humans have been reported [36–39], where human alveolar macrophages were found to be significantly larger (*p* < 0.05) than for rats (21.2 μm vs 13.1 μm). It has been hypothesized that the phagocytic capabilities of these cells may differ between species due to inherent or acquired differences in alveolar macrophage size [36]. Additionally, humans possess a greater number of alveolar macrophages in the airways in comparison with rats [37]. These differences are likely to reflect the different respiratory species - as obligate nose breathers, rats are exposed to fewer airway particulates than human lungs [37]. Interspecies differences in inflammatory pathway mediators (NO, TNFα) and phagocytic activity have also been demonstrated between rat and human alveolar macrophages [38, 39]. Characterising the phenotypic and functional responses of foamy alveolar macrophages in rat and human is key to developing robust models for accurate inhaled safety prediction in humans.

As alveolar macrophage responses are complex, a single parameter assay is unlikely to be adequately descriptive of the different types of ‘foamy’ alveolar macrophage responses to drugs modulating airway biology. The multi-parameter assay approach described in this paper permits a more detailed characterization of how inhaled drugs impact airway macrophage responses and how these develop with duration of and concentration of exposure. A major challenge of this approach is devising an appropriate analysis format for the copious individual cell data generated by the high content assay technique. This is particularly important when using this technique as a screening tool for the prediction of drug safety. It has been demonstrated previously that hierarchical clustering of similar multi-parameter data sets has proven an effective and unbiased method of grouping compounds that generate similar phenotypic profiles in cells *in vitro* [40, 41]. Future work will focus on employing the methodologies described to a broader set of reference compounds that are established to induce alveolar macrophage responses to ascertain if these techniques are sufficiently sensitive to discern different ‘foamy’ alveolar macrophage phenotypes.

## Conclusion

A high content analysis methodology was successfully developed and applied to characterizing the morphology and lipid content of NR8383 and U937 cells and macrophages from rat lungs. Cell vacuolisation and lipid content were similar between rat and human models for cells in culture. All alveolar lung macrophage cell models responded to challenge with amiodarone, a known inducer of phospholipidosis, but detection of these required analysis of cell sub-populations (the proportion of cells with elevated vacuolation or lipid content) rather than average population data which was insensitive to the changes observed. Future work will focus on establishing morphological and lipid profiles of *in vitro* alveolar macrophages following exposure to a broad range of compounds known to induce macrophage responses (and selected controls), and comparing these to effects *in vivo*, and complementary pathway analysis to explore the foamy macrophage phenotype. This work has shown that the multi-parameter methodology has the potential to offer an early non-clinical screening tool to predict the safety of candidate inhaled medicines.

## ACKNOWLEDGMENTS AND DISCLOSURES

The authors acknowledge funding by NC3R for Crack-It Challenge 14: Inhalation Translation, Award NC/C013203/1 “Differentiating alveolar macrophage responses to inhaled medicines”. The Inhalation Translation consortium is formed by GSK, Envigo, King’s College London, University of Hertfordshire and the National Physical Laboratory.

## Abbreviations

BAL: Bronchoalveolar lavage
DMEM/F12: Dulbecco’s modified Eagle’s medium/Ham’s F12
DMSO: Dimethyl sulphoxide
FBS: Fetal bovine serum
FCCP: Carbonyl cyanide-4-(trifluoromethoxy)phenylhydrazone
K-F12: Kaighn’s modified Ham’s F12
NO: Nitric oxide
NR8383: Rat alveolar macrophage cell line
PBS: Phosphate buffered saline
PMA: Phorbol myristate acetate
RPMI-1640: Roswell Park Memoral Institute medium
SD: Standard deviation
TNFα: Tumour necrosis factor alpha
TX-100: Triton X-100
U937: Human lung monocyte cell line
